# Molecular Evaluation of *Chlamydia trachomatis* Infection and Its Association with Tumor Necrosis Factor-α Polymorphism in Recurrent Spontaneous Abortions

**DOI:** 10.4314/ejhs.v31i6.18

**Published:** 2021-11

**Authors:** Fatemeh Darvishi Kolor, Jalil Vand Yousefi, Reza Ranjbar

**Affiliations:** 1 Department of Microbiology, College of Basic Science, Karaj Branch, Islamic Azad University, Alborz, Iran; 2 Shila Medical Diagnostic Laboratory Tehran Iran; 3 Molecular Biology Research Center, Systems Biology and Poisonings Institute, Baqiyatallah University of Medical Sciences, Tehran, Iran

**Keywords:** Chlamydia trachomatis, Recurrent spontaneous abortions (RSA), TNF α-308 polymorphism, PCR

## Abstract

**Background:**

The rate of infections in the intensive care units (ICUs) is rising, mainly because of the increasing use of invasive procedures. Several bacterial infections and host genetic backgrounds, including TNFα SNP polymorphisms, play important roles in recurrent spontaneous abortions (RSA). So, this study aimed to evaluate C. trachomatis infection and its relation with TNFα -308 and -238 polymorphism for early detection and treatment of RSA.

**Methods:**

The blood samples were taken from 63 Iranian women with a history of RSA and 59 ethnically matched healthy controls with at least two successful pregnancies and no history of abortion. Then, DNA was extracted from all samples and detection of C. trachomatis and TNFα -308 and -238 polymorphism was determined by multiplex amplification-refractory mutation system (ARMS)-PCR. Finally, the statistical analysis to detection C. trachomatis and host genetic roles in RSA were analyzed by Epi Info ^TM^ software by X^2^ test.

**Results:**

C. trachomatis was detected in 22 and 3% of the RSA and the control group, respectively. Moreover, in comparison with healthy controls, C. trachomatis infection was closely correlated with TNFα -308 genotypes, whereas no significant association was observed between TNFα -238G/A and RSA. In addition, statistical analysis of TNFα -308 genotypes showed that the frequency of genotype AA was higher in patients with C. trachomatis infections than healthy individuals and the difference was statistically significant.

**Conclusions:**

This study demonstrated that molecular analysis of TNF -308 genotypes is important in early detection and treatment of RSA with C. trachomatis infection.

## Introduction

Spontaneous abortion is one of the most common complications of pregnancy. It occurs in one in five pregnancies and can have considerable physiological and psychological implications for the patient (1). Recurrent spontaneous abortions (RSA), defined as a pregnancy failure occurring before 24 weeks of gestation more than two or three times according to most definitions, is a fertility defect encountered in 1–5% of the patients (2, 3). It is also associated with high health care costs. RSA can be caused by genetic, endocrine, anatomic, immunologic factors, and infection(4). There is evidence that potentially preventable infections may account for up to 15% of early miscarriages and up to 66% of late miscarriages (5–7) *Chlamydia trachomatis* is the leading cause of bacterial sexually transmitted diseases (STDs) in humans(8). According to 2008 WHO report, there are 105 million new cases of STDs due to *C. trachomatis* each year, and the infection rate has been increasing steadily (9–11). However, because patients with *C. trachomatis* urogenital infections often do not exhibit any symptoms (75 to 90% of patients), they remain undiagnosed and untreated (12). This can lead to tubal factor infertility, miscarriage, or ectopic pregnancy, which is a life-threatening condition (13–16).

On other hands, as a host genetic background, tumor necrosis factor-α (TNF-α) is a potent cytokine produced by mononuclear phagocytes, natural killer (NK) cells, and antigen-stimulated T-cells (17–20). It has often been associated with increased risk for RSA. Circulating levels of TNF-α are higher both in animals and humans with a miscarriage compared to those with a successful pregnancy, suggesting that this cytokine is exclusively harmful to pregnancy (21–23). The TNF-α is located within the human leukocyte antigen class III region in chromosome 6p21.3. The production of TNF-α can be controlled by genetic polymorphisms, especially in the promoter regions such as −238; −308; and −863 and especially −308 G/A is known to cause an altered promoter activity, resulting in an increased production of TNF-α cytokine in blood (24–26). Therefore, this study aims to evaluate *C. trachomatis* infection and its association with TNFα -308 and -238 polymorphism for early detection and treatment of RSA.

## Materials and Methods

**Study design and setting**: This cross-sectional study was performed on blood samples obtained from 63 Iranian women experiencing RSA and 59 ethnically matched healthy controls. Informed consent was obtained from all the subjects to carry out molecular analyses, and approval of the institutional ethical committee was obtained. Detailed pedigree analyses and in-depth evaluation of the clinical reports were undertaken in all the subjects. Patients with anatomical abnormalities, acquired or hereditary thrombosis, hormonal disorders, abnormal thyroid function, hyperprolactinemia, erythroblastosis fetalis (Rh disease), chromosomal aberrations, toxoplasmosis, other infections such as Rubella, Cytomegalovirus, Herpes simplex virus (TORCH) infections were excluded from the molecular study. The control group including healthy women with two or more children were selected for statistical comparisons.

**Molecular detection of *C. trachomatis***: Genomic DNA was extracted from peripheral blood leukocytes by DNA extraction kit ((Cinagen Inc. (Tehran, Iran) using the ‘blood and body fluid’ spin protocol. The extracted DNA was stored at -20 °C for further analysis. PCR was carried out the detection of *C. trachomatis* using the KL1 and KL2 primers (Table 1) (27). The PCR amplification including an initial denaturation process at 95°C for 10 min, followed by 35 cycles at 95°C for 1 min, 58 °C for 56 secs, and 72°C for 58 sec. The final extension was performed at 72°C for 8 min (27). The PCR fragments were run on 2% agarose gel with safe stain dye (CinnaGen Co. Iran) and finally were visualized in gel documentation system.

**Table 1:** Nucleotide sequences of the primer sets

Name	Sequence	Size (bp)	Reference
KL1 primer	5′- TCCGGAGCGAGTTACGAAGA -3′	20 Mer	
KL2 Primer	5′- AATCAATGCCCGGGATTGGT -3′	20 Mer	

**TNF-α -308 and -238 genotyping by Multiplex ARMS-PCR**: Multiplex amplification-refractory mutation system (ARMS)-PCR analysis was used to detect TNF-α polymorphism at positions -308 and -238. Three primers (one common, one specific for the mutant, and one specific for the wild-type allele) were used to detect each polymorphism (17). The used PCR -primers are listed in Table 2. The multiplex ARMS -PCR was carried out using 100 ng of DNA extracted from samples with specific primers and standard conditions of PCR amplification. The PCR amplification including an initial denaturation process at 94°C for 5 min, followed by 35 cycles at 94°C for 60 secs, 55 °C for 60 secs, and 72°C for 35 sec. The final extension was performed at 72°C for 10 min in thermocycler(17). The electrophoresis of the PCR fragments (140 bp and 210 bp) was undertaken at 50 V for 1.5 h on 2% agarose gel and was visualized by sybergreen staining under UV trans-illuminator.

**Table 2:** Nucleotide sequences of the primer sets for TNF-α polymorphism

Name	Sequence
-308G	5′-ACCCTGGAGGCTGAACCCCGTCTC-3′
-308A	5′-ACCCTGGAGGCTGAACCCCGTCTT-3′
-238G	5′- ACCCTGGAGGCTGAACCCCGTCTC -3′
-238A	5′- ACCCTGGAGGCTGAACCCCGTCTT -3′
Common primer	5′- GCCCCTCCCAGTTCTAGTTCTATC -3′

**Statistical analysis**: Statistical analysis of data was performed by Epi Info package program. Associations between genotypes, virulence markers and clinical diseases were assessed. Statistical analyses to detect *C. trachomatis* and TNF-α polymorphism roles in RSA were performed by χ2 (Chi-square test) and p-values below 0.05 were considered statistically significant. Expected genotype frequencies were calculated from the allele frequencies under the assumption of Hardy-Weinberg equilibrium.

## Results

Sixty-three cases of RSA and 59 healthy control women with two or more children were genotyped for the TNF-α-238 and TNF-α-308 promoter polymorphisms to find the association of these genotypes or alleles with *C. trachomatis*. The age range of the study sample was from 20 to 35 years. The mean age of maternal and controls was 25.9 and 26.4 years, respectively. The mean miscarriages were 2.4 and the mean gestational age was 3.7 months. Interestingly, the presence of RSA was significantly associated with family history.

**Figure 1:**
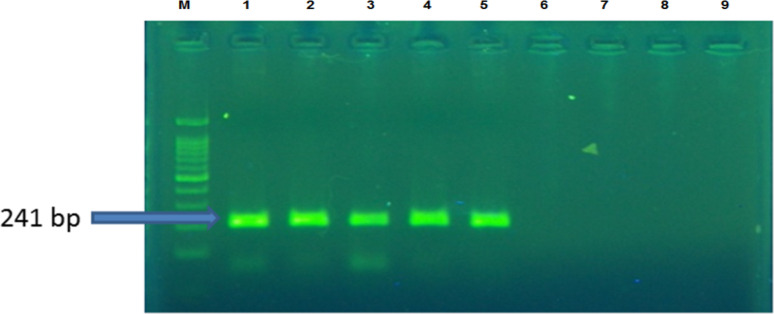
Electrophoresogram of PCR products, Lane M, 100-bp ladder; lanes 2, 3, 3 and 5 samples C. trachomatis positive; lanes 6,7 and 8 samples C. trachomatis negative; lanes 9 sample control negative

***C. trachomatis* infection:** According to molecular analysis, the prevalence of *C. trachomatis* infection was significantly higher in RSA as compared to controls. Out of 63 cases of RSA, *C. trachomatis* was found in 14 (22.22%) while only 2 (3.38%) of controls were positive for *C. trachomatis*, which was found to be significant (Chi-square test: 14.81, P-value = 0.0001) (Figure 1).

**Figure 2:**
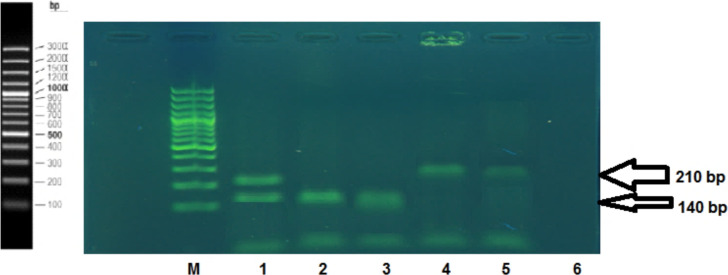
Electrophoresis of ARMS- PCR products for TNF-α (-308 and -238) genotyping Lane M, 100-bp laddr; *lane 1 positive for -308 AA and -238 GG genotypes; lane 2 and 3 positive for -308 AA and -308 GG genotypes; lane 4 and 5 positive for -238 AA and 238 GG genotypes; lane 6 negative control*

**TNF-α polymorphism genotyping**: Out of 63 cases of RSA, the occurrence of GG, GA, and AA genotypes in TNF-α-308 promoter polymorphism were 0.19, 0.46, and 0.35, respectively, in contrast, the occurrence of GG, GA, and AA genotypes in controls cases were 0.64, 0.36, and 0.18 respectively (Figure.2). The frequency for G allele in RSA and controls cases was 0.42 and 0.69 and 0.57 and 0.31 for A allele, respectively. There was statistically significant difference in genotype distribution of alleles in RSA and controls cases. Statistical analysis revealed that TNF-α-308A/A genotypes in the present sample are associated with recurrent miscarriages (Chi-square value for trend = 24.10 and P< 0.0001). Individuals carrying the AA homozygous genotype have a 6.6-times greater risk of presenting with RSA than an individual with the GG homozygous genotype.

On the other hand, in cases with RSA, the distribution of TNF-α-238G/A promoter polymorphism was found to be 0.17, 0.21, and 0.62 for GG, GA, AA genotypes, respectively, whereas those for standard control, were calculated to be 0.13, 0.24, and 0.63 for GG, GA, and AA, respectively. The allele frequency for G in affected and control cases was 0.28 and 0.25, respectively, while for A allele was 0.72 and 0.75. There was no report of prevalent allele in a particular group. Thus, there was no significant statistical difference in genotype the distribution and allele frequency between recurrent miscarriage cases and controls. The distribution of Alleles and genotype frequencies are indicated in Table 3. TNF-α-238G/A promoter polymorphism in the present case-control study does not show any association with the recurrent miscarriages (Chi-square value for trend = 0.37 and P > 0.05).

**Table 3:** Frequency of TNFα-308 genotypes in RSA samples with *C. trachomatis* (+) and *C. trachomatis* (-)

Genotypes	*C. trachomatis* (+)	*C. trachomatis* (-)	Odds Ratio
**GG**	21	43	1.00
**AG**	29	27	1.60
**AA**	50	20	5.11

(Chi-square value for trend = 19.33 and P < 0.0001)

**Association *C. trachomatis* with TNFα -308 polymorphism**: The frequency of TNFα -308 AA genotyping among RSA with and without *C. trachomatis* infection was 50 and 20%, respectively. (Chi-square value for trend = 19.33 and P< 0.0001). The Frequency of TNFα -308 genotypes in RSA samples with and without *C. trachomatis* infection are shown in Table 3. Individuals carrying the TNFα -308 AA homozygous genotype with *C. trachomatis* infection have a 5.11-times more significant risk of presenting with RSA than an individual with the GG homozygous genotype. The TNFα -308 AA genotype was strongly associated with *C. trachomatis* (+) samples compared with *C. trachomatis* (-) samples. Additionally, the presence of RSA was significantly associated with family history and ages. Furthermore, molecular analysis of TNFα -308 AA genotype and *C. trachomatis* are important in the early detection and treatment of RSA.

## Discussion

RSA is a common disorder and represents a major concern for reproductive problems affects approximately one in 300 pregnancies (1–3). Until now, various factors such as genetic, endocrine, anatomic, immunologic, and microbiologic factors have been identified that influence miscarriage. However, the exact underlying etiology in up to 50% of RSA patients remains undetermined (5–7, 9). The incidence of RSA is controlled by genetic factors, and genetic polymorphisms associated with poor pregnancy outcomes. Some studies have shown that cytokines play an important role in the maintenance of pregnancy by modulating the immune system (10, 11, 13–15).

It is also detected that enhanced uterine expression of pro-inflammatory cytokines such as tumor necrosis factor (TNF)-α and interferon-gamma (IFN-γ), interleukin (IL)-1β has been associated with embryo loss (16, 17, 19, 25). Anti-inflammatory cytokines such as IL-6 and IL-10 are considered essential for maintaining a normal pregnancy. The TNF-α has antitumor activity in various tumor cell lines, including breast cancer cell lines (20–22). The TNF-α arrests cell cycle transition from G1 to S phase in mammary carcinoma cells and induces apoptosis in tumor cells. The TNF-α -308G>A and -238G>A polymorphisms are associated with altered TNF expression in vivo and in vitro(23–25).

Our study investigated the association between two common TNF-α gene polymorphisms with RSA. The results show that polymorphism in the TNF-α -308 is associated with RSA, but other polymorphisms have no association with RSA. In this regard, previous studies of the association of TNF-α polymorphisms with RSA risk revealed that TNF-α -308G>A was associated with RSA in caucasian women (22–25). In addition, Lee et al., revealed that the *TNF-α* -1031T>C and *TNF-α* - 238G>A variants increased the risk of RSA (28). The TNF α polymorphisms in different ethnic populations investigated and detected a weak association between the A variant and RSA risk in Iranian populations(24). In addition, several studies failed to find the association between the common polymorphisms in the TNF-α gene and RSA risk(22–25). One possible reason behind this pattern of results could be that -308G/A polymorphism was more impactful than - 238G/A on TNF-α gene expression and protein production, thereby possibly contributing to RSA risk. Moreover, stratification by geographic position, the polymorphism of -308G/A was significantly associated with RSA risk for Asians rather than non-Asians .Zhang et al., found TNF-α-308G/A, −238G/A polymorphisms were not associated with the risk of RPL in the overall population(26). Also, Stavros et al., reported that the TNF-α 238 and TNF-α 308 variants were both identified in RPL and control groups, but there was no statistically significant association between both groups(29). While, Li et al., in a meta-analysis study, revealed that the TNF-α-308G/A polymorphism is associated with susceptibility to RSA, especially in Asian populations, suggesting that TNF-α may play a role in RSA susceptibility (25). There is evidence that potentially preventable infections may account for up to 15% of early miscarriages and up to 66% of late miscarriages (5). *C. trachomatis*, an obligate intracellular bacterium, is the most common sexually transmitted bacterial disease worldwide. The prevalence of the disease is high, estimated at 105 million new cases in 2008 worldwide (6, 7, 9). Though in women, it is often asymptomatic, untreated *C. trachomatis* infection can result in mucopurulent cervicitis, acute urethral syndrome and pelvic inflammatory disease (PID). *C. trachomatis* infection is a known risk factor for ectopic pregnancy and preterm birth (10, 11, 13, 14). This study found that *C. trachomatis* infection might play an effective role in the incidence of RSA. Meantime, the prevalence of *C. trachomatis* infection was significantly higher in RSA than control. These results suggested that *C. trachomatis* infection might play a critical role in RSA.

In a study, 21.3% of miscarriage cases were shown to have persistent *C. trachomatis* infection as determined by levels of sera IgA against *C. trachomatis* major outer membrane protein (7). The authors suggest an association between persistent *C. trachomatis* infection and miscarriage. However, these cases were compared only against patients with tubal infertility and not uninfected pregnant women. Shemer-Avni et al. detected that TNF-a is able to inhibit replication of *C. trachomatis* in vitro (9).

Prasad et al. investigated role of Th1/Th2/Th17 cytokines in the immunopathogenesis of spontaneous abortion in *C. trachomatis* (Ct)-positive first-trimester aborters (16). They found IFN-γ, TNF-α, IL-2, IL-6 and IL-17A cytokines were significantly increased in spontaneous abortion ((SA) group and recurrent miscarriage (RM) group (Ctinfected) versus controls. Furthermore, IFN-γ, TNF-α, IL-6, IL-17A cytokines were significantly elevated in Ct-positive RM group versus *Chlamydia*-infected SA group. Williams et al. demonstrated the TNF-alpha was produced in vivo during *C. trachomatis* infection and plays a role in host defense (10). The lack of clinical characterization of *C. trachomatis infection* may be mentioned as one of the main limitations of the current study.

In conclusion, this study showed significant associations between *C. trachomatis* and its association with TNFα -308 polymorphism in RSA patients. In particular, the TNF-α-308G/A polymorphism and *C. trachomatis* are associated with susceptibility to RSA. Detection of *C. trachomatis* and TNFα -308 AA genotypes play roles in early detection and early treatment of RSA. Because TNF-α gene frequencies differ among ethnic groups, there is a need for large and heterogeneous population-based genetic studies to confirm our findings and hypotheses.
